# Divergent CMV and EBV co-infection impact in adult and pediatric hematopoietic stem cell transplantation recipients

**DOI:** 10.3389/fcimb.2026.1793798

**Published:** 2026-10-14

**Authors:** Osama Alsmadi, Hasan Hashem, Khalid Halahleh, Jude Jarrah, Yazan Talab, Iyad Sultan, Maysa Al-Hussaini

**Affiliations:** 1 Department of Cell Therapy and Applied Genomics, King Hussein Cancer Center, Amman, Jordan; 2 Faculty of Allied Medical Sciences, Applied Science University, Amman, Jordan; 3 Department of Internal Medicine, King Hussein Cancer Center, Amman, Jordan; 4 Artificial Intelligence and Data Innovation Office, King Hussein Cancer Center, Amman, Jordan

**Keywords:** adult, co-infection, EBV_CMV, GvHD (graft-versus-host disease), pediatrics, stem cell transplant (SCT)

## Abstract

**Background:**

Cytomegalovirus (CMV) and Epstein-Barr virus (EBV) reactivations are major complications after hematopoietic stem cell transplantation (HCT), but co-infection epidemiology and outcomes in combined adult-pediatric cohorts remain poorly defined.

**Materials and methods:**

This retrospective single-center study analyzed 941 consecutive HCT recipients (2015–2023). CMV and EBV were quantified by whole-blood qPCR (positivity ≥153 and ≥433 copies/mL, respectively). CMV testing was weekly until day +100 post-HSCT; EBV testing followed clinical indication at the treating physician’s discretion. IgG serostatus is not routinely tested at KHCC for post-transplant monitoring. Acknowledging some missing values, we evaluated the incidence, risk factors, and survival impact of CMV/EBV co-infection using chi-square tests, Kaplan-Meier analysis, and multivariable logistic and Cox proportional-hazards regression.

**Results:**

Among 941 recipients (56.5% adult, 43.5% pediatric), adults received 89.1% of CMV tests (838/941) and 76.6% of CMV infections (105/137), whereas pediatric patients accounted for 59.8% of EBV infections (76/127); EBV testing covered 65.5% (616/941). Co-infection incidence was 7.6% (70/916). Co-infection was significantly associated with allogeneic transplant (p=0.004), haploidentical donor (p=0.010), reduced-intensity conditioning (p=0.017), and acute graft-versus-host disease (aGVHD) (p=0.028), but not with malignancy status (54/70 [77.1%] vs. 577/760 [75.9%] malignant; p=0.819); haploidentical (vs. related) donor type remained independent on multivariable analysis (adjusted OR 2.01, 95% CI 1.18–3.41, p=0.0098). CMV monoinfection was associated with adult age and haploidentical transplantation; EBV monoinfection with pediatric age, non-malignant disease, and aGVHD. Pediatric 3-year overall survival (OS) was superior (78.4% vs. 64.6%; median follow-up 41.8 months). The difference in 3-year OS between co-infected (67.3%) and non-co-infected (70.9%) patients did not reach statistical significance (Kaplan–Meier log-rank p = 0.4046; crude mortality by Pearson chi-square, p = 0.286); chronic GVHD was the only independent predictor of OS (adjusted HR 0.016, 95% CI 0.002–0.150, p=0.0003).

**Conclusion:**

CMV and EBV post-HCT exhibit distinct, age-associated risk profiles, and co-infection defines a high-risk phenotype linked to intensive immunosuppression and aGVHD. Three-year OS did not differ significantly between groups; given the observational design, selection bias from non-uniform EBV testing, and possible type II error, this should not be taken as evidence that pre-emptive strategies eliminate the survival impact of co-infection. Findings support age- and risk-adapted viral monitoring.

## Introduction

Allogeneic hematopoietic stem cell transplantation (allo-HCT) is a curative therapy for hematologic malignancies, but its success is often hampered by viral reactivations, especially in the immunocompromised host. Cytomegalovirus (CMV) and Epstein-Barr virus (EBV) are two of the most consequential pathogens post-transplant. CMV reactivation is a well-established cause of morbidity and non-relapse mortality, particularly in adult recipients (Ivana et al., 2022). In contrast, EBV is a frequent driver of post-transplant lymphoproliferative disorder (PTLD), a serious complication with a higher incidence in pediatric populations (Dierickx and Habermann, 2018). CMV is a critical viral pathogen in allo-HCT recipients, with reactivation rates ranging from 66% in European regions, and as high as 90% in Eastern Mediterranean population (AlQahtani et al., 2024). The global seroprevalence of CMV is estimated at 83% (95%UI: 78-88) in the general population (Zuhair et al., 2019). CMV infection can lead to a spectrum of clinical manifestations, including CMV disease, which may present as fever, malaise, and cytopenias. Severe manifestations can involve the lungs, gastrointestinal tract, and central nervous system (Spadea et al., 2023). CMV pneumonia, in particular, is associated with high morbidity and mortality, necessitating prompt recognition and treatment. The clinical course of CMV infection can also involve chronic infection, persisting for months post-transplant (Fowler et al., 2022). EBV on the other hand, a member of the herpesvirus family, establishes lifelong latency in infected individuals. In the context of allo-HCT, reactivation of EBV can lead to serious complications, primarily post-transplant lymphoproliferative disorder (PTLD). PTLD occurs due to uncontrolled proliferation of B cells infected with EBV, leading to symptoms such as fever, lymphadenopathy, and organomegaly (Zhou et al., 2020). The risk of PTLD, while rare, is particularly heightened in seronegative recipients or those receiving grafts from EBV-positive donors, highlighting the importance of vigilant monitoring. Apart from PTLD, EBV reactivation can contribute to immune dysregulation, exacerbating graft-versus-host disease (GVHD) and increasing susceptibility to opportunistic infections (Hickey et al., 2020).

While the independent clinical courses of CMV and EBV are well-documented, their simultaneous reactivation presents a complex clinical scenario that is less understood. The immune response to one herpesvirus can influence the control of the other; for instance, CMV-driven T-cell reconstitution may impair immune surveillance of EBV, potentially elevating the risk of PTLD (Hickey et al., 2020). As noted by Zhou et al. (2020) and Li et al. (2022), co-reactivation of CMV and EBV has been associated with poor prognosis after allogeneic stem cell transplantation. This interplay suggests that co-infection could lead to worse outcomes than either infection alone. More recent data have added important distinction to this picture: increasing use of letermovir CMV prophylaxis since 2017–2018 has been associated with a compensatory increase in EBV reactivation after haploidentical transplantation (Kong et al. (2024)), while a recent 5-year single-center cohort study found that isolated, sustained CMV replication, rather than transient CMV/EBV co-reactivation, was the dominant independent predictor of late (3- and 5-year) mortality after allo-HCT (Li et al. (2026)), suggesting the clinical significance of co-reactivation may be time-dependent and modulated by contemporary antiviral strategies. However, the epidemiology, risk factors, and overall survival impact of CMV/EBV co-infection across a combined adult and pediatric cohort remain poorly characterized, with existing literature focusing on these age groups or viruses in isolation. Anderson-Smits et al. (2020) systematically reviewed coinfection rates and clinical outcomes, highlighting the need for further research in this area.

As a retrospective analysis, we aim to elucidate the clinical characteristics and outcomes associated with EBV and CMV co-infection among HCT recipients. This gap in knowledge is critical, especially with the increasing use of haploidentical transplants, which are known to carry a high risk of viral complications (Li et al., 2022). Therefore, we conducted this retrospective study at King Hussein Cancer Center (KHCC) to define the incidence, identify the independent predictors, and evaluate the survival impact of CMV/EBV co-infection in a real-world cohort of both adult and pediatric HCT patients.

## Materials and methods

### Study design and population

A retrospective study and analysis for 941 consecutive HCT recipients at KHCC (2015–2023). Inclusion criteria: First HCT for malignant/non-malignant disease, ≥30 days follow-up post-HCT, and two age groups including pediatric (<18 years) and adults (≥18 years). Both allogeneic (76.1%) and autologous (23.9%) transplant recipients were included in the overall cohort. Because autologous HCT carries minimal CMV/EBV reactivation risk in the absence of alloimmune suppression, co-infection and survival analyses included all patients with available CMV and EBV testing results regardless of transplant type; among the 916 patients included in the co-infection analysis, 709 (77.4%) underwent allogeneic and 207 (22.6%) underwent autologous transplantation, and among the 70 patients with CMV/EBV co-infection, 64 (91.4%) were allogeneic and 6 (8.6%) were autologous recipients. Transplant type (allogeneic vs. autologous) was itself entered as a candidate risk factor in the co-infection association analysis (**Supplementary Table 1**) rather than autologous recipients being excluded *a priori*. We acknowledge that pooling allogeneic and autologous recipients, who differ markedly in reactivation risk, may dilute the observed co-infection incidence; this is discussed as a limitation below.

### CMV and EBV testing

CMV and EBV DNA loads were measured using quantitative Qiagen real-time polymerase chain reaction (qPCR) kits (Qiagen artus CMV RG PCR Kit and Qiagen artus EBV RG PCR Kit, respectively) on whole blood EDTA samples. The CMV assay had a lower limit of detection of 153 copies/mL; values ≥153 copies/mL were considered positive. The EBV assay had a lower limit of detection of 433 copies/mL; values ≥433 copies/mL were considered positive. CMV testing was performed frequently from engraftment until day +100 post-HCT, or longer if clinically indicated. EBV testing was performed based on clinical suspicion at the treating physician’s discretion; routine weekly EBV screening was not performed. Although pre-transplant CMV and EBV serostatus (IgG antibodies) are not reported in this analysis, they are mainly employed to guide donor selection and, consequently, are not incorporated into post-transplantation monitoring protocols at KHCC. We acknowledge donor-recipient serostatus as one of the strongest known predictors of post-transplant viral reactivation; its unavailability is discussed as a limitation below. Viral reactivation post-HCT was defined as new or increased detectable viral genomic copies after transplantation in patients with pre-existing latency; persistence was defined as continuous detectable low-level viremia without significant fluctuation.

### Study outcomes

The study aims to compare CMV/EBV incidence and outcomes between adult and pediatric HCT recipients, identify risk factors for coinfection and its association with GVHD, and assess survival impacts in a real-world Middle Eastern cohort.

### Statistical analysis

Univariate analysis was done using Chi-square/Fisher’s exact tests (categorical) and t-tests/Mann-Whitney (continuous), in addition to multivariable analysis using logistic regression for infection predictors, and Cox regression for survival analysis (Kaplan-Meier with log-rank test). SAS v9.4 statistical package (two-sided *p* < 0.05) was used for the statistical analysis. Variables measured at the patient level (including malignancy status) were analyzed against co-infection status at the patient level. Categorical associations, including the comparison of vital status (alive versus dead) at last follow-up, were assessed using the Pearson chi-square test and represent crude, time-independent proportions; overall survival was analyzed separately using the Kaplan–Meier method with the log-rank test, which incorporates follow-up time and censoring. p-values derived from these two approaches address different questions and are not directly comparable. Multivariable models used complete-case analysis; variables significant at p<0.05 in univariate analysis were entered as candidate predictors, and variables with sparse categories that precluded model convergence were excluded, as detailed alongside each model in the Results and **Supplementary Material**. Reporting follows STROBE guidance for observational studies, including transparent reporting of missing data by variable throughout the Results and **Supplementary Tables**.

### Ethics statement

This project was reviewed and approved by the research office and IRB Ethics committee under the protocol number 24 KHCC 144. An informed consent waiver was approved by the IRB upon data anonymization.

## Results

### Clinical categorical variables and demographics

Our analysis details a significant and clinically relevant cohort of 941 patients who underwent hematopoietic cell transplantation (HCT), characterized by a male predominance (64.6%) and a well-represented mix of both adult (56.5%) and pediatric (43.5%) patients. This demographic balance enhances the generalizability of our findings across a broad age range. The most salient feature of the cohort is the high prevalence of underlying malignant diseases (76.0%), with Leukemia being the single most common diagnosis (48.7%), followed by Lymphoma (17.2%), while non-malignant conditions accounted for the remaining 24.0% of cases (**Figure 1**; **Supplementary Table 2**).

**Figure 1:**
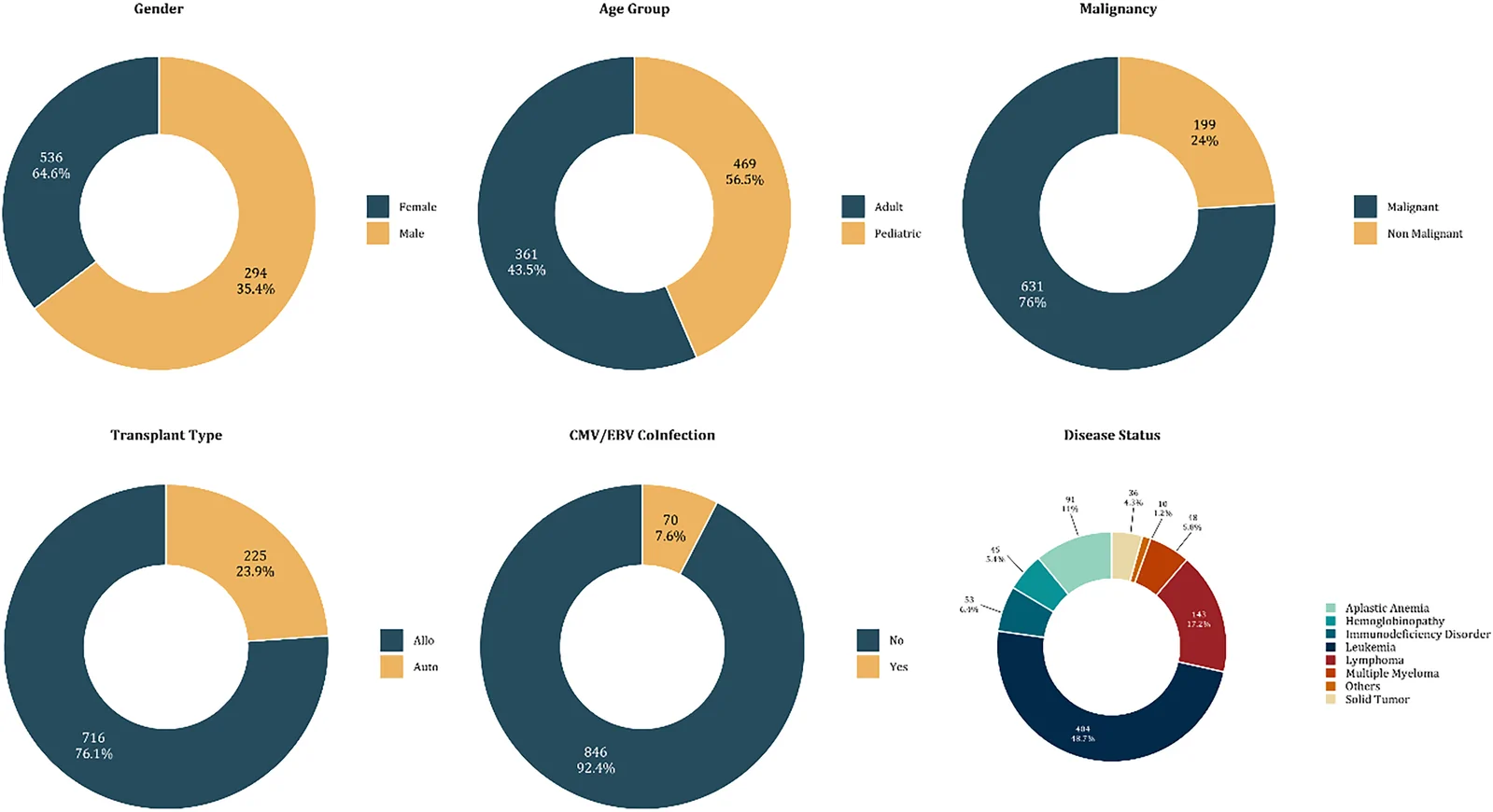
Distribution of categorical variables, including gender, age, malignancy status, disease subtype, transplant type, and CMV/EBV co-infection status. Distribution of baseline categorical variables in the study cohort (N = 941). Gender: male, n = 536 (64.6%); female, n = 294 (35.4%). Age group: adult, n = 469 (56.5%); pediatric, n = 361 (43.5%). Malignancy status: malignant, n = 631 (76.0%); non-malignant, n = 199 (24.0%). Transplant type: allogeneic, n = 716 (76.1%); autologous, n = 225 (23.9%). CMV/EBV co-infection: positive, n = 70 (7.6%); negative, n = 846 (92.4%). Disease status: leukemia, n = 404 (48.7%); lymphoma, n = 143 (17.2%); aplastic anemia, n = 91 (11.0%); immunodeficiency disorder, n = 53 (6.4%); multiple myeloma, n = 48 (5.8%); hemoglobinopathy, n = 45 (5.4%); solid tumor, n = 36 (4.3%); other diagnoses, n = 10 (1.2%). Percentages are calculated from the full cohort and correspond to **Supplementary Table 2**.

This predominance of malignancies fundamentally shaped the clinical management strategy, directly explaining the preferential use of allogeneic HCT (76.1%) over autologous procedures (23.9%) and the corresponding reliance on myeloablative conditioning regimens (72.4%). While allogeneic transplants are often necessary for curative intent in malignancies, they carry a well-established higher risk of immunosuppression and viral reactivation compared to autologous procedures. This treatment profile establishes a patient population at significant risk for opportunistic infections, making the subsequent virological data critically important. As Safdar (Safdar et al., 2011) noted, patients with hematologic malignancies are particularly vulnerable to CMV and EBV due to immunosuppressive therapies.

### Disparities in viral testing practices

Within this high-risk context, our analysis reveals a crucial disparity in screening practices for key opportunistic pathogens. The standard of care cytomegalovirus (CMV) testing was performed on the vast majority of the cohort (89.1%, 838/941), whereas Epstein-Barr virus (EBV) testing was conducted less frequently on 65.5% (616/941) of all patients. This is a critical finding, as both CMV and EBV are major drivers of post-transplant morbidity and mortality. Among the 830 patients included in the patient-level demographic analysis, 469 (56.5%) were adults and 361 (43.5%) were pediatric patients. CMV testing was performed in 838 of 941 (89.1%) cases (testing instances), whereas EBV testing was performed in 616 of 941 cases (65.5%); these are case-level denominators, distinct from the 830 patient-level demographic denominator, and are reported separately here to avoid the ambiguity. The prevalence of confirmed infection also differed by age group. Adult patients comprised the majority of CMV infections (76.6%, 105/137). Conversely, pediatric patients accounted for the majority of EBV infections (59.8%, 76/127). These findings suggest that the risk and surveillance for CMV and EBV differ meaningfully between adult and pediatric transplant recipients, which could inform targeted monitoring strategies.

Given the intense immunosuppression associated with allogeneic transplants and myeloablative conditioning, this population remains at persistent risk for viral reactivation throughout the post-transplant period. The identified incidence of CMV/EBV co-infection was relatively low (7.6%, 70/916 after excluding missing values). However, this finding must be interpreted with considerable caution because EBV testing was performed in only 65.5% of patients, and testing was often clinically indicated rather than universal, which likely underestimates the true co-infection rate. Metafuni et al. (2021) similarly reported on CMV and EBV reactivation after allogeneic stem cell transplantation, emphasizing the importance of routine monitoring.

### Associations between CMV/EBV co-infection and clinical variables

Our univariate analysis reveals that CMV/EBV co-infection is significantly clustered within a distinct high-risk patient profile. Out of 11 variables tested, four demonstrated a statistically significant association (p-value ≤ 0.05) with co-infection, all of which are intrinsically linked to the degree of iatrogenic immunosuppression and alloreactive immune dysregulation (**Supplementary Table 1**).

Underlying disease category was not associated with co-infection. Because CMV/EBV co-infection was ascertained at the case level whereas malignancy status is a patient-level characteristic, this association was evaluated at the patient level. Among the 70 co-infected patients, 54 (77.1%) had a malignant and 16 (22.9%) a non-malignant underlying disease, compared with 577 (75.9%) and 183 (24.1%) respectively among the 760 non-co-infected patients (Pearson chi-square, p = 0.819). Malignant disease therefore reflects the composition of the transplanted cohort (76.0% overall) rather than an independent driver of co-infection risk. Ding et al. (2021) reported that EBV and CMV reactivation after allogeneic HCT in patients with non-Hodgkin lymphoma had a significant impact on outcomes; that cohort was, however, restricted to a single malignant diagnosis and therefore does not address whether malignancy itself predisposes to co-reactivation.

The type of transplant, by contrast, was strongly associated with co-infection (p = 0.004). Amongst the allogeneic transplanted patients, 91.4% of co-infected patients received allografts compared to 76.2% in the non-co-infected group. Delving deeper into allogeneic transplant specifics, the donor type was also a significant factor (p = 0.010). Haploidentical transplants were overrepresented in the co-infected group (50.0% vs. 33.0%).

In contrast to myeloablative conditioned patients, prevalence of co-infection was relatively more significant (41.4%, p = 0.017) in patients who received Reduced Intensity Conditioning (RIC). The most significant clinical consequence of this immunologic landscape was the development of Acute Graft-versus-Host Disease (GVHD), which was significantly more frequent in co-infected patients (p = 0.028). Over half (51.6%) of the co-infected patients developed Acute GVHD, compared to 37.5% of the non-co-infected patients. Iftikhar et al. (2023) reported similar findings, demonstrating that CMV infection post-allogeneic stem cell transplantation is associated with increased GVHD risk in populations with high seropositivity.

### Multivariable analysis of infection predictors and survival

For adjusted statistical estimates, multivariable logistic regression and Cox proportional-hazards regression were performed. Full model details, including excluded variables and the reasons for exclusion, are provided in the **Supplementary Material**.

In multivariable logistic regression for CMV/EBV co-infection (N = 709; candidate predictors: transplant type, donor type, conditioning intensity, GVHD prophylaxis, acute GVHD), haploidentical (vs. related) donor type was independently associated with co-infection (adjusted OR 2.01, 95% CI 1.18–3.41, p=0.0098); acute GVHD showed a non-significant trend (adjusted OR 1.66, 95% CI 0.98–2.79, p=0.058) (**Supplementary Table 4**).

For CMV monoinfection (N = 709; candidate predictors: age group, malignancy status, donor type), adult age (adjusted OR 3.60, 95% CI 2.15–6.04, p<0.0001) and haploidentical donor type (adjusted OR 3.20, 95% CI 2.08–4.93, p<0.0001) remained independently associated with CMV infection (**Supplementary Table 5**).

For EBV monoinfection (N = 709; candidate predictors: age group, malignancy status, acute GVHD), pediatric age (adjusted OR 0.51 for adult vs. pediatric, 95% CI 0.33–0.81, p=0.0040), non-malignant disease (adjusted OR 0.60 for malignant vs. non-malignant, 95% CI 0.38–0.94, p=0.0268), and acute GVHD (adjusted OR 1.64, 95% CI 1.08–2.49, p=0.0194) were independently associated with EBV infection (**Supplementary Table 6**).

In multivariable Cox regression for overall survival (N = 226; candidate predictors: gender, age group, malignancy status, stem cell source, chronic GVHD), chronic GVHD was the only variable independently associated with survival (adjusted HR 0.016, 95% CI 0.002–0.150, p=0.0003); age group (adjusted HR 1.69, 95% CI 0.98–2.92, p=0.059) and malignancy status (adjusted HR 2.14, 95% CI 0.89–5.11, p=0.088) showed non-significant trends (**Supplementary Table 7**). Complete-case multivariable Cox regression could not be performed for the CMV/EBV co-infection variable itself due to sample size constraints after listwise deletion; this is noted as a limitation below.

### Time-to-event analysis

Time-to-event analysis was performed relative to either the date of sample receipt (viral detection) or the date of HCT. For intervals measured from sample receipt to GVHD onset, statistics indicated that viral detection typically preceded both acute and chronic GVHD for most patients. Notable variability was observed, particularly in pediatric cohorts, which exhibited wider standard deviations and more extreme minimum/maximum values (data not shown). Analysis of time from sample receipt to HCT confirmed that viral detection frequently occurred pre-transplantation (median CMV: -38.5 days; median EBV: -72.0 days), with pediatric patients showing greater variability in this interval, especially for EBV.

When measured from HCT date to GVHD onset, the timelines were more consistent. The median time from HCT to acute GVHD was similar between CMV and EBV cohorts (33 vs. 30 days), with adult patients developing acute GVHD slightly later than pediatric patients in both groups. The median time from HCT to chronic GVHD was substantially longer (CMV: 149 days; EBV: 174 days), with less pronounced age-related differences.

In contrast to these strong clinical associations, several factors showed no significant link to co-infection. The distribution of males and females (p = 0.835) and the proportion of adult and pediatric patients (p = 0.911) were nearly identical between groups.

Although a higher crude mortality rate was observed in the co-infected group (35.7% vs. 29.6%), this difference did not reach statistical significance in the patient-level association analysis (Pearson chi-square, p = 0.286). This finding should be interpreted cautiously, as the lack of statistical significance may reflect sample size limitations or the effectiveness of pre-emptive therapy rather than a true absence of effect. Data on chronic GVHD were unreliable due to a high number of missing values, precluding any meaningful conclusion.

### Associations between CMV-only infection and clinical variables

The analysis of CMV infection reveals a risk profile that is both distinct and highly instructive (**Supplementary Table 3**). A paramount finding is that while allogeneic transplantation is a well-known risk factor for CMV, the specific type of allograft is more critical than the broad category itself. In our cohort, merely undergoing an allogeneic (vs. autologous) transplant was not a significant risk factor for CMV infection alone. Instead, when examining allogeneic transplant recipients, the source of the graft played a critical role; transplants from haploidentical donors accounted for over half (53.0%) of all CMV infections. Miyao et al. (2021) compared transplantation outcomes after foscarnet and ganciclovir administration as first-line anti-CMV preemptive therapy, highlighting the importance of antiviral strategies in this high-risk population.

Furthermore, the analysis revealed a highly statistically significant association (p < 0.001) between CMV infection and patient age. Patients with a CMV infection were, on average, seven years older than those without, with a median age of 30 years in the CMV-positive group compared to 20 years in the CMV-negative group.

The link to the underlying disease was also highly significant (p < 0.001), with CMV infection being much more common in patients with a malignant disease, who comprised 84.7% of the CMV-positive group. Interestingly, and in contrast to the co-infection analysis, CMV infection in this cohort showed no significant association with the development of GVHD or with ultimate patient survival.

For comparative context, a parallel analysis revealed a highly significant association (p < 0.001) between EBV infection and patient age, but in the opposite direction. Patients with an EBV infection were significantly younger, with a median age of 15 years compared to 24 years in the EBV-negative group.

### Associations between EBV-only infection and clinical variables

Our analysis confirms that specific patient and transplant characteristics define a high-risk profile for EBV reactivation (**Supplementary Table 8**) following HCT. A primary finding is the significant divergence in risk based on age and underlying disease. EBV infection was significantly more prevalent in pediatric patients, who comprised 59.8% of the EBV-positive group compared to 40.2% for adults (p<0.001). Furthermore, a higher infection incidence was observed in patients with non-malignant underlying diseases (35.4% of EBV-positive patients) than in those with malignant diseases (p=0.001). Tsushima et al. (2024) reported similar clinical characteristics and outcomes of EBV viral load after allogeneic hematopoietic stem cell transplantation.

A critical clinical finding was the significant link between EBV infection and the subsequent development of acute Graft-versus-Host Disease (aGVHD). Patients with EBV infection had a significantly higher rate of aGVHD (47.9%) compared to those without EBV infection (37.0%; p=0.027). Post-transplant lymphoproliferative disorder (PTLD), though mostly linked to solid organ transplants, is an extremely rare event at KHCC, and has no clinical impact on our EBV-positive stem cell transplant patients, and further has caused no EBV-related deaths. This suggests most of our post-HCT EBV viremia cases were due to reactivation of latent virus rather than persistence of a pre-existing active infection. Lindsay et al. (2021) provided an update on EBV post-transplant lymphoproliferative disorder management and outcomes, confirming that early detection and intervention are critical. Conversely, no statistically significant associations were found between EBV infection and several other variables, including gender, the broad type of transplant (allogeneic vs. autologous), donor type, conditioning intensity, or overall patient survival.

### Mortalities associated with age

Median follow-up among surviving patients (N = 580) was 41.8 months (mean 45.9 months; range, 0.6–239.7 months; SD 33.4 months) (**Supplementary Table 9**). A significant and stark disparity in survival outcomes was observed between pediatric and adult patients undergoing stem cell transplantation. Pediatric patients demonstrated a markedly higher three-year overall survival rate of 78.4% (95% C.I.: 73.7%, 82.8%) compared to 64.6% (95% C.I.: 59.8%, 69.3%) in adult patients (**Table 1**; **Figure 2**).

**Table 1:** Three-year overall survival in transplanted adult and pediatric patients.

Pediatric/adult	Three-year OS rate (95% C.I.)
Adult	64.6% (59.8-69.3%)
Pediatric	78.4% (73.7-82.8%)

**Figure 2:**
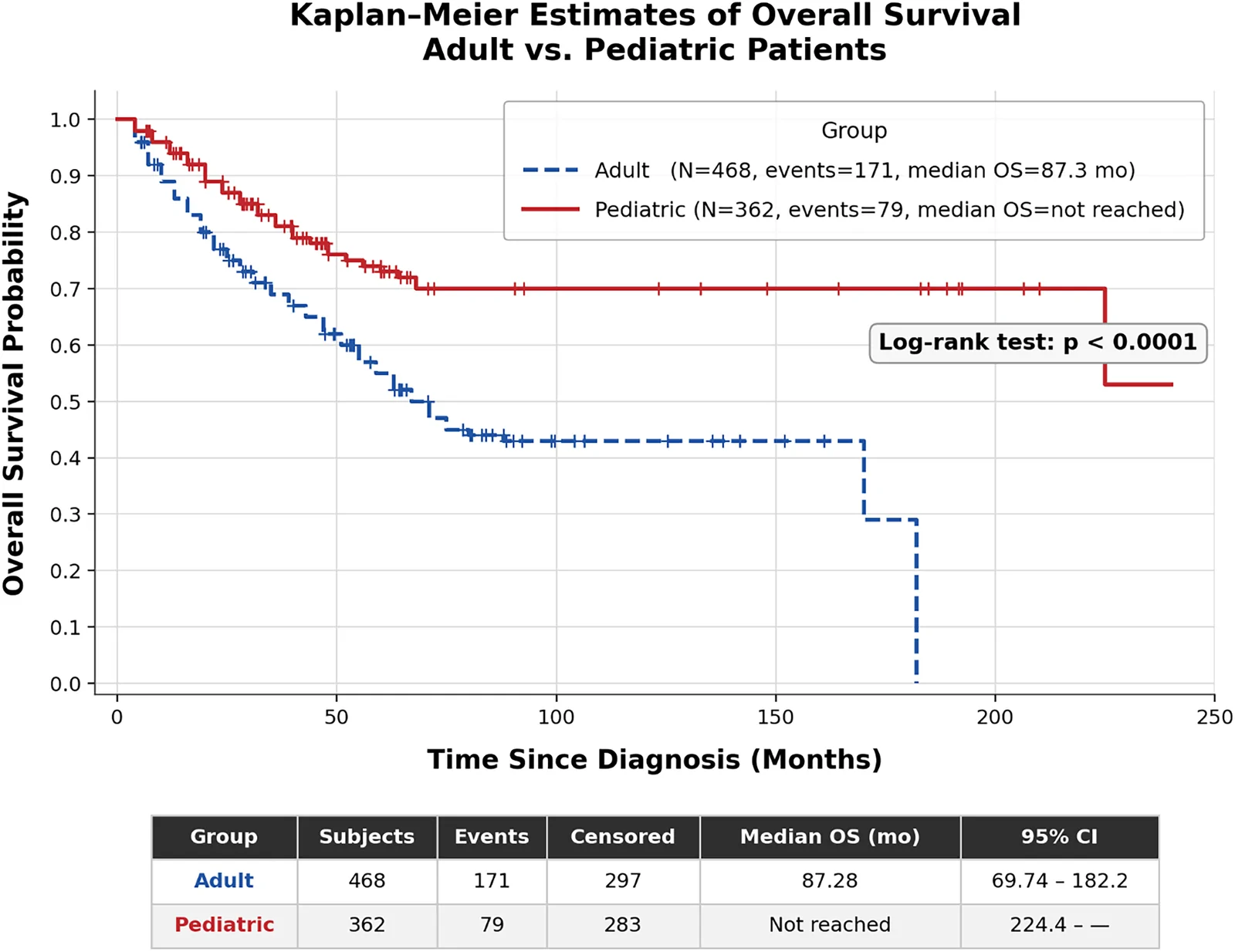
Kaplan-Meier analysis of age comparing adults to pediatrics. Pediatric patients showed significantly better overall survival than adults (log-rank p < 0.0001). Median OS was 87.28 months in adults versus not reached in pediatric patients, with the pediatric survival curve consistently higher throughout follow-up. The pediatric curve plateaued near 70% survival for an extended period before a late decline to ~53%, while adult survival fell progressively to near 0% by ~180 months. These findings indicate age group is a strong prognostic factor for overall survival in this cohort. Overall survival was calculated from the date of transplantation to the date of death or last available follow-up; patients alive at last follow-up were censored at that date. Survival curves were compared using the log-rank test.

### Mortalities associated with CMV/EBV co-infection

The comparison of three-year overall survival (OS) rates between patients with and without CMV/EBV co-infection reveals a nuanced picture of its clinical impact. Analysis shows that patients without co-infection had a three-year OS of 70.9%, compared to 67.3% in the co-infected group. The difference in 3-year OS between co-infected and non-co-infected patients did not reach statistical significance (Kaplan–Meier with log-rank test, p = 0.4046) (**Table 2**; **Figure 3**). This time-to-event comparison is distinct from the crude mortality comparison reported in **Supplementary Table 1**, in which the proportion of patients recorded as deceased at last follow-up did not differ significantly between groups (35.7% vs. 29.6%; Pearson chi-square, p = 0.286). The two p-values derive from different analyses, a time-independent association test and a time-to-event analysis incorporating censoring, and are therefore not contradictory.

**Table 2:** Three-year overall survival between co-infected and non-co-infected patients.

CMV/EBV co-infection	Three-year OS rate (95% C.I.)
No	70.9% (67.3-74.4%)
Yes	67.3% (53.6-79.6%)

**Figure 3:**
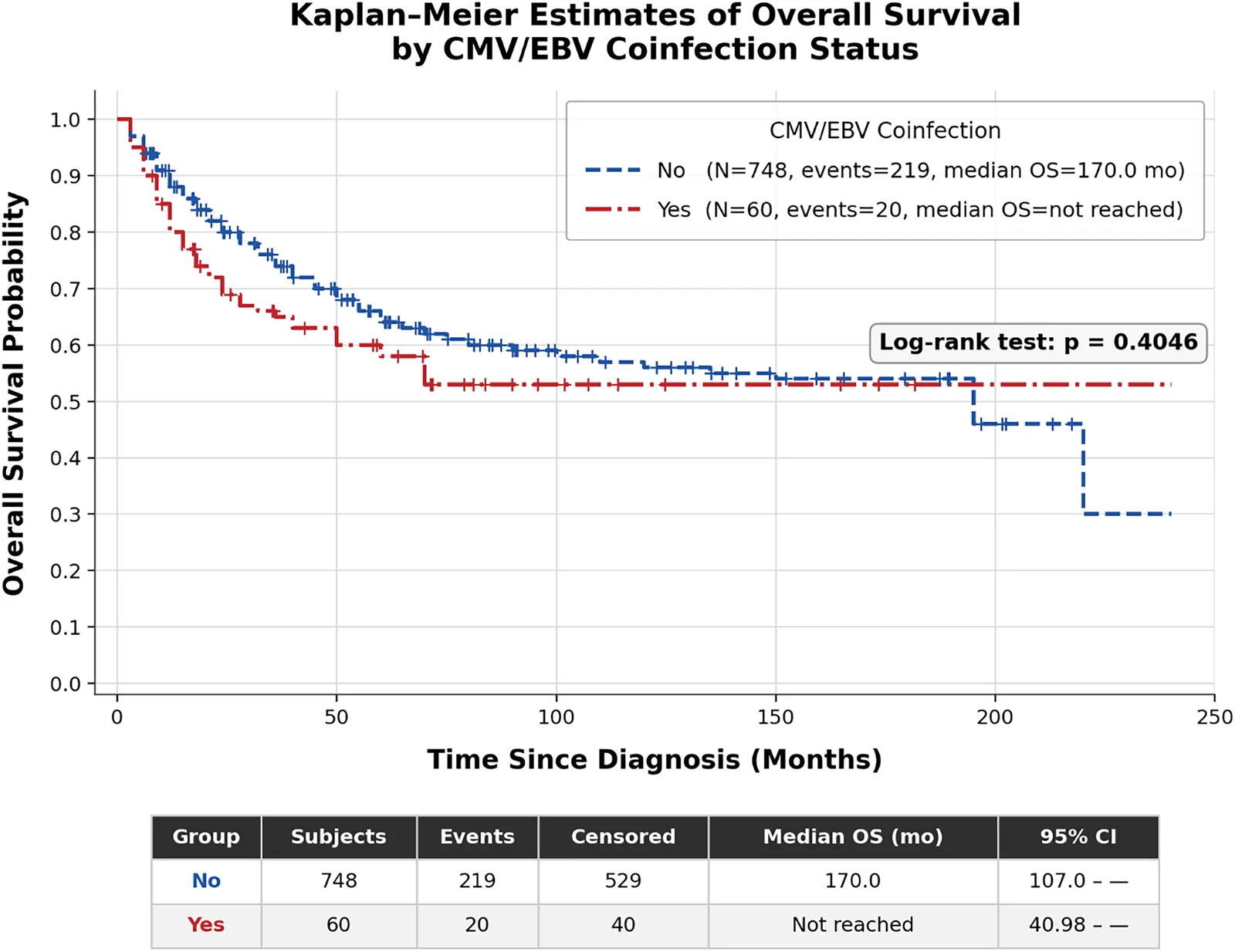
Kaplan-Meier analysis of CMV/EBV co-infection relative to no infection state. There was no significant difference in overall survival between patients with and without CMV/EBV coinfection (log-rank p = 0.4046). The curves overlapped substantially, with the “Yes” group (N = 60) showing a somewhat steeper early decline but converging with the “No” group (N = 748) by around 70–90 months, both plateauing near 53–58% thereafter. Median OS was 170.0 months in the “No” group versus not reached in the “Yes” group, but given the non-significant p-value, this difference is not statistically meaningful. Overall, CMV/EBV coinfection status does not appear to be a significant prognostic factor for survival in this cohort. Overall survival was calculated from the date of transplantation to the date of death or last available follow-up; patients alive at last follow-up were censored at that date. Survival curves were compared using the log-rank test.

## Discussion

This comprehensive analysis of 941 HCT patients provides several important insights into the distinct clinical landscapes of CMV and EBV reactivation. Our findings demonstrate that these two herpesviruses are driven by divergent risk profiles: CMV reactivation predominates in older patients and those undergoing haploidentical transplantation, while EBV reactivation preferentially affects pediatric recipients and those with non-malignant diseases, with significant association with aGVHD.

### Comparison with previous studies

Our finding that CMV reactivation is more common in adults (76.6% of CMV infections) aligns with Anderson-Smits et al. (2020), who reported higher CMV seroprevalence and reactivation rates in older populations. Conversely, our observation that EBV infection predominates in pediatric patients (59.8%) compared to adults (40.2%) is consistent with Styczynski et al (Styczynski et al., 2013; Styczynski et al., 2016), who noted that pediatric HCT recipients have higher rates of EBV-related PTLD due to more frequent EBV naivety and altered immune reconstitution. The co-infection rate of 7.6% in our cohort is comparable to the 6-10% range reported by Li et al. (2022) in their meta-analysis of CMV/EBV co-reactivation. However, direct comparisons are limited by heterogeneous testing protocols across studies. Consistent with our observation of divergent, age-associated risk profiles for CMV and EBV, a more recent 5-year single-center cohort study found that isolated, sustained CMV replication, rather than transient CMV/EBV co-reactivation, was the dominant independent predictor of late (3- and 5-year) mortality after allo-HCT (Li et al. (2026); this offers a possible explanation for why our own co-infection survival comparison, drawn from a cohort with shorter median follow-up (41.8 months), did not reach statistical significance, and underscores that our non-significant finding should not be read as evidence that co-reactivation is clinically inconsequential over longer follow-up.

The association between co-infection and aGVHD (51.6% vs. 37.5%, p=0.028) mirrors the bidirectional relationship described by Gooptu and Koreth (2020), where viral reactivation and GVHD create a pro-inflammatory feedback loop. Unlike previous reports by Mariotti et al. (2022), who found CMV reactivation independently predicted mortality in haploidentical transplants, our survival analysis did not demonstrate a statistically significant mortality difference between co-infected and non-co-infected patients. This discrepancy may reflect differences in pre-emptive therapy protocols, sample size, or patient populations. Malard et al. (2023) recently reviewed acute graft-versus-host disease, emphasizing the complex interplay between viral infections and GVHD pathogenesis.

An important contextual factor not captured in our dataset is letermovir prophylaxis, which received regulatory approval for CMV prevention in CMV-seropositive adult allo-HCT recipients in 2017–2018 and has since become standard of care in many centers (Marty et al. (2017). A large real-world meta-analysis confirmed that letermovir prophylaxis substantially reduces clinically significant CMV infection and CMV disease, with lower all-cause and non-relapse mortality beyond day 200 post-transplant (Vyas et al. (2022). Because our study period (2015–2023) spans the pre- and post-letermovir era, and letermovir use was not systematically recorded in this dataset, secular changes in CMV prophylaxis practice may confound comparisons of CMV incidence and outcomes across calendar time, and could partly explain why CMV monoinfection was not independently associated with mortality in our cohort. Notably, emerging evidence suggests that CMV suppression in the letermovir era may be associated with a compensatory increase in EBV reactivation, particularly after haploidentical transplantation (Kong et al. (2024), a dynamic that merits attention in future co-infection studies. Updated international consensus recommendations further emphasize risk-adapted, extended CMV surveillance beyond day 100 in high-risk recipients, consistent with the age- and risk-adapted monitoring strategy we propose (Ljungman et al. (2025).

### Limitations and clinical implications

Several limitations warrant acknowledgment, reported here in line with STROBE guidance for observational studies. First, EBV testing was not performed uniformly across the cohort; only 65.5% of patients received EBV testing, and this was often clinically indicated rather than protocol-driven, introducing substantial selection bias and likely underestimating the true co-infection rate. Second, the inclusion of both allogeneic and autologous recipients, who differ markedly in reactivation risk and in the intensity with which they were tested, may dilute the observed co-infection incidence; this heterogeneity should be considered when interpreting our estimates, although transplant type was itself modeled as a candidate risk factor rather than used as an exclusion criterion. Third, and importantly, pre-transplant donor-recipient CMV and EBV serostatus (IgG), among the strongest known predictors of post-transplant viral reactivation, was not available for this analysis; this precluded serostatus-based risk stratification and is a limitation in this study. Fourth, this retrospective dataset did not capture letermovir prophylaxis use, because this drug is not part of the standards of care at KHCC. Our study period (2015–2023) coincides with the broader adoption of letermovir prophylaxis internationally, although letermovir was not used at our institution during this time. Secular changes in CMV prophylaxis practice at other centers may limit comparability of our CMV incidence and outcome estimates with more recent published cohorts, and may partly explain why CMV monoinfection was not independently associated with mortality in our analysis. Fifth, multivariable logistic regression and Cox proportional-hazards models adjusting for clinically relevant confounders are now reported in the Results and **Supplementary Material**; these used complete-case analysis, which reduced the effective sample size for some models and required exclusion of predictors with sparse categories (e.g., “no conditioning”, certain GVHD prophylaxis regimens), as detailed alongside each model. A complete-case multivariable Cox model for the co-infection variable itself could not be fit due to sample size constraints and remains an important next step. Sixth, the retrospective single-center design limits generalizability to other populations with different viral epidemiology, testing protocols, or transplantation practices; missing data are reported transparently by variable throughout the Results and **Supplementary Tables**. Seventh, missing data for some variables, particularly chronic GVHD, for which the large majority of records were unknown, substantially reduced statistical power and reliability for those specific analyses, and such findings should be considered exploratory only. Finally, the lack of statistical significance for the co-infection survival comparison should not be overinterpreted; it may reflect type II error related to sample size rather than a true absence of effect, and, as discussed above, may also reflect the relatively short median follow-up (41.8 months) relative to reports suggesting the clinical significance of co-reactivation may only become apparent over longer follow-up.

Despite these limitations, our findings support age- and risk-adapted viral monitoring protocols. Intensified CMV monitoring should be considered for older adults and recipients of haploidentical transplants. Proactive EBV surveillance (including routine weekly qPCR) may be warranted in pediatric patients, those with non-malignant diseases, and patients who develop aGVHD. Tapuchova et al (Ivana et al., 2022). and Annaloro et al. (2020) provided comprehensive reviews on viral infections in HCT, supporting our monitoring recommendations.

### Future directions

It is recommended for future prospective studies to implement universal, protocol-driven EBV testing to accurately define co-infection incidence. Correlation of viral load kinetics with immunosuppressive regimens and immune reconstitution parameters may identify actionable risk periods for pre-emptive therapy. Zhou et al. (2020) and Li et al. (2022) suggested that understanding the mechanisms of co-reactivation could lead to improved therapeutic strategies. Fowler et al. (2022) highlighted the global seroprevalence of CMV, emphasizing the need for region-specific approaches to screening and treatment. Future work at our center should also incorporate donor-recipient serostatus, and extend follow-up to evaluate whether the survival impact of co-reactivation becomes apparent over a longer time horizon, as suggested by recent external cohort data.

## Conclusions

This comprehensive analysis of 941 hematopoietic stem cell transplantation (HCT) patients elucidates the distinct clinical landscapes and impacts of CMV and EBV reactivation. Our findings robustly demonstrate that these two herpesviruses are not monolithic threats but are driven by divergent risk profiles. CMV reactivation is predominantly a challenge in older patients and those undergoing the profoundly immunosuppressive environment of haploidentical transplantation. In stark contrast, EBV reactivation preferentially affects pediatric recipients and those with non-malignant diseases, and is significantly intertwined with the development of acute GVHD. Furthermore, we have delineated a specific high-risk phenotype for CMV/EBV co-infection, characterized by allogeneic and haploidentical transplantation, reduced-intensity conditioning, and acute GVHD, with no association with underlying disease category, with haploidentical donor type remaining independently associated with co-infection after multivariable adjustment, underscoring its genesis from iatrogenic immunosuppression and alloreactive immune dysregulation. Gail et al. (2023) described complex interactions of cellular players in chronic GVHD, providing biological context for our clinical observations.

From an outcomes perspective, while CMV/EBV co-infection is strongly associated with increased morbidity (notably acute GVHD), the observed difference in three-year overall survival did not reach statistical significance, and chronic GVHD, not co-infection status, was the dominant independent predictor of survival in multivariable analysis. This may reflect the efficacy of contemporary pre-emptive monitoring and therapeutic strategies, but could also be influenced by sample size limitations, selection bias due to differential testing, or the relatively short median follow-up (41.8 months) relative to reports suggesting the survival impact of co-reactivation may only emerge over longer follow-up.

In summary, this study provides a refined framework for risk-stratifying HCT recipients for viral complications. Moving forward, clinical vigilance should be tailored: intensified CMV monitoring in older, haploidentical transplant recipients, and proactive EBV surveillance in pediatric patients and those with GVHD.

## Data Availability

The original contributions presented in the study are included in the article/**Supplementary Material**, further inquiries can be directed to the corresponding author/s.

## Supplementary material

Supplementary Table 1 Association between CMV/EBV co-infection and categorical variables. Malignancy status is a patient-level characteristic and was therefore evaluated against co-infection status at the patient level (N = 830; 70 co-infected, 760 non-co-infected). The Alive/Dead row is a crude, time-independent association tested by Pearson chi-square and is distinct from the Kaplan–Meier log-rank comparison of overall survival shown in **Figure 3** (p = 0.4046). p-values ≤0.05 indicate statistical significance. For Acute GVHD, 6 co-infected and 201 non-co-infected patients had missing data; for donor type, 33 patients had missing data.

Supplementary Table 2 Statistical summary for categorical variables (N = 941). Note: All numbers verified against the analysis file provided by the study biostatistician. Percentages calculated from available data with missing values noted.

Supplementary Table 3 Association between CMV Infection and categorical variables (N = 137 CMV-positive). For donor type, 33 CMV-negative patients had missing donor data (autologous transplants excluded from this row).

Supplementary Table 4 Multivariable logistic regression — CMV/EBV co-infection (N = 709). Model included: transplant type, donor type, conditioning intensity, GVHD prophylaxis, acute GVHD. Transplant type, conditioning intensity, and GVHD prophylaxis were excluded from the final model due to sparse categories after complete-case listwise deletion (see main text).

Supplementary Table 5 Multivariable logistic regression — CMV infection (N = 709). *Age (continuous) was not included alongside Pediatric/Adult due to collinearity.*

Supplementary Table 6 Multivariable logistic regression — EBV infection (N = 709). *Age (continuous) was not included alongside Pediatric/Adult due to collinearity.*

Supplementary Table 7 Multivariable Cox regression — overall survival (N = 226). Model included: gender, age group, malignancy status, stem cell source, chronic GVHD. GVHD prophylaxis was excluded due to sparse categories after complete-case listwise deletion.

Supplementary Table 8 Association between EBV infection and categorical variables (N = 127 EBV-positive).

Supplementary Table 9 Follow-up duration among surviving patients.
