# Supplementary material for: Divergent CMV and EBV co-infection impact in adult and pediatric hematopoietic stem cell transplantation recipients
Source: Front Cell Infect Microbiol. 2026 Oct 14;16:1793798. doi: 10.3389/fcimb.2026.1793798 (PMC13713334; doi:10.3389/fcimb.2026.1793798)
Supplement: Supplementary file 1 [file Table1.docx]

**SUPPLEMENTARY MATERIAL**

**Supplementary Table 1: Association between CMV/EBV Co-Infection and categorical variables**

| **Variable** | **Co-Infection: Yes** | **Co-Infection: No** | **Total** | **P-value** |
| --- | --- | --- | --- | --- |
| Female | 24 (34.3%) | 270 (35.5%) | 294 (35.4%) | 0.835 |
| Male | 46 (65.7%) | 490 (64.5%) | 536 (64.6%) |  |
| Adult | 40 (57.1%) | 429 (56.4%) | 469 (56.5%) | 0.911 |
| Pediatric | 30 (42.9%) | 331 (43.6%) | 361 (43.5%) |  |
| Malignant | 54 (77.1%) | 577 (75.9%) | 631 (76.0%) | 0.819 |
| Non-Malignant | 16 (22.9%) | 183 (24.1%) | 199 (24.0%) |  |
| Allo | 64 (91.4%) | 645 (76.2%) | 709 (77.4%) | 0.004 |
| Auto | 6 (8.6%) | 201 (23.8%) | 207 (22.6%) |  |
| Haplo donor | 32 (50.0%) | 213 (33.0%) | 245 (34.6%) | 0.010 |
| Related donor | 30 (46.9%) | 423 (65.6%) | 453 (63.9%) |  |
| Unrelated donor | 2 (3.1%) | 9 (1.4%) | 11 (1.6%) |  |
| Myeloablative | 41 (58.6%) | 620 (73.3%) | 661 (72.2%) | 0.017 |
| Reduced Intensity | 29 (41.4%) | 220 (26.0%) | 249 (27.2%) |  |
| Acute GVHD — Yes | 33 (51.6%) | 242 (37.5%) | 275 (38.8%) | 0.028 |
| Acute GVHD — No | 31 (48.4%) | 403 (62.5%) | 434 (61.2%) |  |
| Alive | 45 (64.3%) | 535 (70.4%) | 580 (69.9%) | 0.286 |
| Dead | 25 (35.7%) | 225 (29.6%) | 250 (30.1%) |  |

*Malignancy status is a patient-level characteristic and was therefore evaluated against co-infection status at the patient level (N=830; 70 co-infected, 760 non-co-infected). The Alive/Dead row is a crude, time-independent association tested by Pearson chi-square and is distinct from the Kaplan–Meier log-rank comparison of overall survival shown in Figure 3 (p = 0.4046). p-values ≤0.05 indicate statistical significance. For Acute GVHD, 6 co-infected and 201 non-co-infected patients had missing data; for donor type, 33 patients had missing data.*

**Supplementary Table 2: Statistical summary for categorical variables (N = 941)**

| **Variable** | **Frequency (%)** |
| --- | --- |
| Gender — Female | 294 (35.4%) |
| Gender — Male | 536 (64.6%) |
| Pediatric/Adult — Adult | 469 (56.5%) |
| Pediatric/Adult — Pediatric | 361 (43.5%) |
| Malignant | 631 (76.0%) |
| Non-Malignant | 199 (24.0%) |
| Type of Transplant — Allo | 716 (76.1%) |
| Type of Transplant — Auto | 225 (23.9%) |
| Conditioning — Myeloablative | 681 (72.4%) |
| Conditioning — Reduced Intensity | 254 (27.0%) |
| Conditioning — No conditioning | 6 (0.6%) |
| CMV Test Done — Yes | 838 (89.1%) |
| CMV Test Done — No | 103 (10.9%) |
| EBV Test Done — Yes | 616 (65.5%) |
| EBV Test Done — No | 325 (34.5%) |
| CMV Positive | 137 (16.4%) |
| CMV/EBV Co-Infection — Yes | 70 (7.6%) |
| CMV/EBV Co-Infection — No | 846 (92.4%) |

*Note: All numbers verified against the analysis file provided by the study biostatistician. Percentages calculated from available data with missing values noted.*

**Supplementary Table 3: Association between CMV Infection and categorical variables (N = 137 CMV-positive)**

| **Variable** | **CMV: Yes** | **CMV: No** | **Total** | **P-value** |
| --- | --- | --- | --- | --- |
| Adult | 105 (76.6%) | 364 (52.5%) | 469 (56.5%) | <0.001 |
| Pediatric | 32 (23.4%) | 329 (47.5%) | 361 (43.5%) |  |
| Malignant | 116 (84.7%) | 515 (74.3%) | 631 (76.0%) | 0.009 |
| Non-Malignant | 21 (15.3%) | 178 (25.7%) | 199 (24.0%) |  |
| Haplo donor | 62 (53.0%) | 183 (30.9%) | 245 (34.6%) | <0.001 |
| Related donor | 53 (45.3%) | 400 (67.6%) | 453 (63.9%) |  |
| Unrelated donor | 2 (1.7%) | 9 (1.5%) | 11 (1.6%) |  |
| Alive | 90 (65.7%) | 490 (70.7%) | 580 (69.9%) | 0.242 |
| Dead | 47 (34.3%) | 203 (29.3%) | 250 (30.1%) |  |

*For donor type, 33 CMV-negative patients had missing donor data (autologous transplants excluded from this row).*

**Supplementary Table 4: Multivariable logistic regression — CMV/EBV Co-Infection (N=709)**

| **Effect** | **Adjusted OR** | **95% CI Lower** | **95% CI Upper** | **P-value** |
| --- | --- | --- | --- | --- |
| Haplo vs Related donor | 2.007 | 1.183 | 3.405 | 0.0098 |
| Unrelated vs Related donor | 3.133 | 0.643 | 15.259 | 0.1575 |
| Acute GVHD: Yes vs No | 1.655 | 0.983 | 2.787 | 0.0583 |

*Model included: transplant type, donor type, conditioning intensity, GVHD prophylaxis, acute GVHD. Transplant type, conditioning intensity, and GVHD prophylaxis were excluded from the final model due to sparse categories after complete-case listwise deletion (see main text).*

**Supplementary Table 5: Multivariable logistic regression — CMV Infection (N=709)**

| **Effect** | **Adjusted OR** | **95% CI Lower** | **95% CI Upper** | **P-value** |
| --- | --- | --- | --- | --- |
| Adult vs Pediatric | 3.602 | 2.148 | 6.039 | <0.0001 |
| Malignant vs Non-Malignant | 1.193 | 0.683 | 2.085 | 0.5353 |
| Haplo vs Related donor | 3.202 | 2.079 | 4.931 | <0.0001 |
| Unrelated vs Related donor | 3.683 | 0.728 | 18.633 | 0.1150 |

*Age (continuous) was not included alongside Pediatric/Adult due to collinearity.*

**Supplementary Table 6: Multivariable logistic regression — EBV Infection (N=709)**

| **Effect** | **Adjusted OR** | **95% CI Lower** | **95% CI Upper** | **P-value** |
| --- | --- | --- | --- | --- |
| Adult vs Pediatric | 0.514 | 0.326 | 0.808 | 0.0040 |
| Malignant vs Non-Malignant | 0.597 | 0.378 | 0.942 | 0.0268 |
| Acute GVHD: Yes vs No | 1.642 | 1.083 | 2.489 | 0.0194 |

*Age (continuous) was not included alongside Pediatric/Adult due to collinearity.*

**Supplementary Table 7: Multivariable Cox regression — Overall Survival (N=226)**

| **Effect** | **Adjusted HR** | **95% CI Lower** | **95% CI Upper** | **P-value** |
| --- | --- | --- | --- | --- |
| Male vs Female | 1.146 | 0.683 | 1.922 | 0.6063 |
| Adult vs Pediatric | 1.692 | 0.980 | 2.921 | 0.0592 |
| Malignant vs Non-Malignant | 2.135 | 0.893 | 5.109 | 0.0883 |
| Chronic GVHD: Yes vs No | 0.016 | 0.002 | 0.150 | 0.0003 |

*Model included: gender, age group, malignancy status, stem cell source, chronic GVHD. GVHD prophylaxis was excluded due to sparse categories after complete-case listwise deletion.*

**Supplementary Table 8: Association between EBV Infection and categorical variables (N = 127 EBV-positive)**

| **Variable** | **EBV: Yes** | **EBV: No** | **Total** | **P-value** |
| --- | --- | --- | --- | --- |
| Adult | 51 (40.2%) | 418 (59.5%) | 469 (56.5%) | <0.001 |
| Pediatric | 76 (59.8%) | 285 (40.5%) | 361 (43.5%) |  |
| Malignant | 82 (64.6%) | 549 (78.1%) | 631 (76.0%) | 0.001 |
| Non-Malignant | 45 (35.4%) | 154 (21.9%) | 199 (24.0%) |  |
| Acute GVHD — Yes | 56 (47.9%) | 219 (37.0%) | 275 (38.8%) | 0.027 |
| Acute GVHD — No | 61 (52.1%) | 373 (63.0%) | 434 (61.2%) |  |
| Alive | 84 (66.1%) | 496 (70.6%) | 580 (69.9%) | 0.318 |
| Dead | 43 (33.9%) | 207 (29.4%) | 250 (30.1%) |  |

**Supplementary Table 9: Follow-up duration among surviving patients**

| **N** | **Mean (mo)** | **Median (mo)** | **Min** | **Max** | **Range** | **SD** |
| --- | --- | --- | --- | --- | --- | --- |
| 580 | 45.94 | 41.79 | 0.62 | 239.67 | 239.05 | 33.37 |
